# Immunological Response Against SARS-COV-2 After BNT162b2 Vaccine Administration Is Impaired in Allogeneic but Not in Autologous Stem Cell Transplant Recipients

**DOI:** 10.3389/fonc.2021.737300

**Published:** 2021-09-06

**Authors:** Martina Chiarucci, Sara Paolasini, Alessandro Isidori, Barbara Guiducci, Federica Loscocco, Maria Capalbo, Giuseppe Visani

**Affiliations:** Hematology & Hematopoietic Stem Cell Transplant Center, Azienda Ospedaliera Marche Nord, Pesaro, Italy

**Keywords:** SARS-COV2, immune response, vaccine, allogeneic, autologous, transplant

## Abstract

The efficacy of Covid-19 vaccine in hematopoietic stem cell transplantation (HSCT) recipients is still unknown. We planned a prospective study to evaluate the immune response after the administration of Covid-19 vaccine in HSCT recipients. Fifty patients previously submitted to HSCT (38 autologous and 12 allogeneic) received the mRNA-based SARS-CoV-2 vaccine BNT162b2 (Pfizer-BioNTech). Serum samples of all patients were tested for SARS-CoV-2 IgG against the Spike glycoprotein, 30 days after the second dose of vaccine. Antibody response was compared to a control group of 45 healthy subjects. Of the 50 patients tested, 12 did not develop any antibody response, including 6 patients undergoing autologous (16%) and 6 allogeneic HSCT (50**%**). Cyclosporine administration in allogeneic recipients and prior administration of Rituximab in the autologous setting correlated with lower antibody titers (p < 0.0003 and p=0.000, respectively). Flow cytometry of peripheral blood samples, performed 30 days after the vaccination, showed a significant correlation between the antibody response to Sars-COV2 and an increased number in CD19+ B lymphocytes (p = 0.0003) and CD56+ natural killer (NK) cells (p = 0.00). In conclusion, prior Rituximab before autologous HSCT and cyclosporine administration after allogeneic HSCT negatively affected the antibody response to Sars-COV2 vaccine, possibly due to their immunosuppressive action on CD20 +B cells and T cells, respectively. The correlation between seroconversion to Sars-COV2 and higher number of CD19 + B cells and CD56+ NK cells, suggests a central role for B and NK cells in the development of COVID-19 immunity after vaccination with a mRNA-based platform.

## Introduction

Novel coronavirus disease 2019 (COVID-19) caused by severe acute respiratory syndrome virus (SARS-CoV-2) has determined a global health care crisis. Most of the end-organ complications that characterize severe COVID-19 are linked to a deregulated immune response that follows SARS-CoV-2 infection. Phase 3 trials of COVID-19 mRNA-based vaccines showed an efficacy greater than 90% in preventing symptomatic infection, following two doses administered 3 to 4 weeks apart. Furthermore, the risk of severe illness from COVID-19 has been reported to be lowered by more than 90% after vaccination (1, 2).

The cumulative observational evidence suggests that hosts with altered immunity including, in particular, hematopoietic stem cell transplantation (HSCT) recipients, may be at elevated risk of complications and death due to SARS-CoV-2 infection (3). This patient population, accordingly, needs special attention, as infections represent a main concern after HSCT and are a major cause of transplant related mortality. Up to now, data are lacking on the immune response to SARS-CoV-2 infection in patients receiving chronic immunosuppressive therapies. In addition, it is still unclear if mRNA-based COVID-19 vaccines are effective in patients submitted to HSCT, and there are several other unanswered questions about COVID-19 vaccinations in patients on immunosuppressive agents (4). We thus planned to study the immune response after the administration of mRNA-based SARS-CoV-2 vaccine BNT162b2 (Pfizer-BioNTech) in HSCT recipients.

## Materials and Methods

On March 2021, as suggested by GITMO and SIE indications (5), 50 HSCT recipients (38 autologous HSCT and 12 allogeneic HSCT, all transplanted in our Unit) (**Table 1**) were vaccinated with the mRNA-based SARS-CoV-2 vaccine BNT162b2 (Pfizer-BioNTech), according to the standard protocol (two doses of 30 µg administered three weeks apart) (6). None of these patients had previously reported SARS-CoV-2-infections; 50% of allo-HSCT patients were receiving immunosuppressive medication(s) at the time of vaccination.

**Table 1:** Patients characteristics and antibody response to Sars-COV2 vaccination.

	N° (%)	Production of Anti Sars-COV2 IgG antibody (% of patients)	p-value
All patients	50 (100)	76%	
**Sex**			
Male	28 (56)	78%	ns
Female	22 (44)	76%	
**Age (years)**			
≤60	27 (54)	66%	0.0003
>60	23 (46)	87%	
**HSCT**			
Autologous	38 (76)	87%	0.0000
Allogeneic	12 (24)	50%	
**Diagnosis (auto-HSCT)**			
Multiple myeloma	22/38 (58%)	95%	NA
Non-Hodgkin lymphoma	14/38 (37%)	71%	
Hodgkin lymphoma	2/38 (5%)	100%	
**Diagnosis (allo-HSCT)**			
Acute myeloid leukemia	7/12 (59%)	57%	NA
Acute lymphoblastic leukemia	4/12 (33%)	25%	
Myelodysplastic Syndrome	1/12 (8%)	100%	
**Prior Rituximab administration (NHL only)**			
Yes	11/14 (78)	54%	0.0000
No	3/14 (22)	100%	
**Time from last Rituximab administration**			
Less than 1 year	5 (45)	20%	0.00
More than 1 year	6 (55)	83%	
**Patients receiving immunosuppressive therapy (allo-HSCT)**			
Yes	6 (50)	17%	0.0003
No	6 (50)	83%	
**GVHD (allo-HSCT)**			
Yes	5 (42)	60%	0.16
No	7 (58)	43%	
**Lymphocyte subpopulation (flow cytometry)**			
B CD19+/mmc (40 patients)			
≤100	17 (42)	65%	0.0003
>100	23 (58)	91%	
T CD4+/mmc (43 patients)			
≤200	17 (39)	70%	0.07
>200	26 (61)	77%	
T CD8+/mmc (43 patients)			
≤300	12 (28)	83%	0.001
>300	31 (72)	71%	
NK CD56+/mmc (41 patients)			
≤100	10 (24)	40%	0.0000
>100	31 (76)	87%	

HSCT, hematopoietic stem cell transplantation; NHL, non-Hodgkin lymphoma; ns, not significant; NA, not applicable.

Serum samples of all patients were tested for SARS-CoV-2 IgG against the Spike glycoprotein, using an approved anti-SARS-CoV-2 IgG CLIA (LIAISON^®^ SARS-CoV-2 TrimericS IgG assay, Diasorin, Saluggia, Italy), 30 days after the second dose of vaccine. According to the manufacturer’s recommendations for the CLIA, an Arbitrary Units per milliliter (AU/mL) ratio of <12.0 was considered to be negative and >15.0 to be positive (borderline 12-15).

In addition, the antibody response was compared to a control group of 45 healthy subjects, vaccinated at the same time and with the same modalities.

Statistical analysis was performed using Chi-square Test to determine the correlation between antibody response and the following variables: age, sex, type of transplant, precedent Rituximab (RTX) therapy, immunosuppressive therapy, lymphocyte subpopulations analyzed by flow cytometry. Results were considered statistically significant when the p-value was below 0.05.

The study was conducted after informed consent, according to the guidelines of the Declaration of Helsinki.

## Results

All HSCT recipients were negative for SARS-CoV-2-IgG prior to vaccination. Median age at the time of transplant was 60 years (range 21-72 years). The first dose of the vaccine was administered 369 days after transplantation (range 5-736 days); all allogeneic HSCT recipients were beyond day 100 from transplant.

Of the 50 evaluable patients, only 12 did not develop any antibody response, 6 of them after autologous HSCT (5 NHL and 1 MM) and 6 after allogeneic HSCT. The median antibody titer in responsive patients was 282 AU/mL (range 68->400).

After the injection of the vaccine, only mild localized pain at the injection-site was frequently reported by study population, whereas severe local reactions, like redness or swelling, were not observed. We observed 2 systemic reactions with fatigue, muscle or joint pain, diarrhea and increased liver enzymes requiring steroid therapy. Thus, overall, the vaccinations were well tolerated.

In patients receiving allo-HSCT, we observed a 42% incidence of mild graft versus host disease (GVHD). Among allogeneic recipients, 6/12 (50%) where under immunosuppressive therapy: 4/6 were on cyclosporine (less than 50 mg/day) and 2/6 on low doses of systemic steroid therapy alone (prednisone <0.5mg/kg/day), by the time of vaccine administration. Patients on cyclosporine at the time of vaccination had lower antibody titers (p < 0.0003) with respect to patients who already stopped immunosuppression. Furthermore, none of the patients still exposed to cyclosporine and within 6 months from allo-HSCT had a protective antibody titer against SARS-CoV-2.

In patients receiving high-dose therapy and auto-HSCT, prior administration of Rituximab was associated with a lower rate of immunization against SARS-CoV-2 (54%) and a significantly lower IgG antibody titer (p = 0.000). Notably, only 20% of patients receiving mRNA-based SARS-CoV-2 vaccine BNT162b2 (Pfizer-BioNTech) within an year from the last dose of rituximab developed a protective antibody titer (p= 0.00).

In allogeneic recipients, cyclosporine administration correlated with lower antibody titers (p < 0.0003); none of the patients within sixth month from HSCT had a protective antibody titer. In autologous setting, prior administration of RTX correlated with lower antibody titer (p = 0.000).

No SARS-CoV-2 infections were reported 12 weeks after the first vaccination in all but 1 autologous patients, who developed mild SARS-Cov2 infection after the first dose.

Flow cytometry was performed on peripheral blood samples, in order to correlate the specific lymphocyte values (using normality cut-off for reference, with the exception of CD4 + T lymphocytes for which we used 200/mmc as threshold value), with the development of antibody response to the vaccine. A significant correlation between the seroconversion to Sars-COV2 and CD19 + B lymphocytes count (p = 0.0003) and CD56 + Natural Killer cell count (p = 0.00) emerged, while there was no correlation with the T lymphocyte subpopulations, neither CD4+ nor CD8+ (lower T CD8+ lymphocyte levels seem to be related with a higher seroconversion rate) (**Table 1**).

## Discussion

In this study we demonstrated that the administration of mRNA-based SARS-CoV-2 vaccine BNT162b2 (Pfizer-BioNTech) in HSCT recipients is safe, and able to generate a protective immune response in approximately 50% of allo-HSCT and 84% of auto-HSCT recipients, respectively. Interestingly, immunization rate in patients receiving auto-HSCT is similar to healthy subjects, and seems to be affected only by precedent Rituximab exposition (in NHL patients) or disease status (progressive disease in a patient with multiple myeloma) (4, 5). The precedent exposition to Rituximab and the concomitant administration of cyclosporine immunosuppressive therapy resulted in antibody response failure to Sars-COV2 vaccine. Rituximab acts on B CD20 + cells inducing cell cycle arrest, direct apoptosis, sensitization to cytotoxic drugs, complement dependent cytotoxicity and antibody dependent cellular cytotoxicity (7). Cyclosporine primarily inhibits the activation cascade necessary for specific immune functions by blocking T-cell-dependent biosynthesis of lymphokines, particularly IL-2, abort intrathymic T-cell development, and inhibits some T-cell-independent B-cell responses (8). Cyclosporine therefore exhibits a direct inhibitory effect on the CD8 + and CD4 + cells stimulated by the vaccine, thus impairing the cellular response induced by vaccine. Notably, all patients receiving cyclosporine did not develop a protective antibody response to the vaccine.

By analyzing lymphocyte subpopulations by flow cytometry, we were able to identify a significant correlation between the seroconversion to Sars-COV2 and CD19+ B lymphocyte and CD56 + Natural Killer cell count, whether no correlation with CD4+ and CD8+ T lymphocyte count and the generation of a protective immune response was found.

This observation was somewhat unexpected, and warrants further investigations, since the m-RNA based BNT162b2 vaccine exerts its function by stimulating the production of neutralizing antibodies and a CD8+/Th1-type CD4+ T cell response (5). We think that CD8+/Th1-type CD4+ T subpopulations should be stimulated by the vaccine independently from their count, and our data showed that there is no correlation between their values after vaccination and the generation of an anti-SARS-COV2 immunity after vaccination. Instead, a correlation emerges between low values of CD19 + B lymphocytes and CD56 + NK cells post vaccine and the response to the vaccine itself, suggesting a possible bystander role of these two cell subgroups in the development of a protective immunity after vaccination. Further studies in a larger cohort of patients are warranted to clarify the role of the different lymphocytes subpopulations in the generation of a long-lasting anti SARS-COV-2 immunization in HSCT recipients.

## Data Availability Statement

The raw data supporting the conclusions of this article will be made available by the authors, without undue reservation.

## Ethics Statement

The studies involving human participants were reviewed and approved by CERM. The patients/participants provided their written informed consent to participate in this study.

## Author Contributions

MC and SP, designed the study and wrote the manuscript. BG and FL collected the data and performed statystical analysis. AI, MC, and GV supervised the study, wrote and approved the final version of the manuscript. All authors contributed to the article and approved the submitted version.

## Conflict of Interest

The authors declare that the research was conducted in the absence of any commercial or financial relationships that could be construed as a potential conflict of interest.

## Publisher’s Note

All claims expressed in this article are solely those of the authors and do not necessarily represent those of their affiliated organizations, or those of the publisher, the editors and the reviewers. Any product that may be evaluated in this article, or claim that may be made by its manufacturer, is not guaranteed or endorsed by the publisher.
